# Comparison of proportions and prognostic impact of pathological complete response between evaluations of representative specimen and total specimen in primary breast cancer after neoadjuvant chemoradiotherapy: an ancillary study of JCOG0306

**DOI:** 10.1007/s10549-024-07408-5

**Published:** 2024-06-27

**Authors:** Tadahiko Shien, Hitoshi Tsuda, Keita Sasaki, Junki Mizusawa, Futoshi Akiyama, Masafumi Kurosumi, Masataka Sawaki, Nobuko Tamura, Kiyo Tanaka, Takahiro Kogawa, Mina Takahashi, Naoki Hayashi, Hirofumi Mukai, Norikazu Masuda, Fumikata Hara, Hiroji Iwata

**Affiliations:** 1 Department of Breast and Endocrine Surgery, Okayama University Hospital, 2-5-1 Shikata-cho, Kita-ku, Okayama, 7008558 Japan; 2 Department of Basic Pathology, National Defense Medical College, Tokorozawa, Japan; 3 JCOG Data Center/Operations Office, National Cancer Center Hospital, Tokyo, Japan; 4 Department of Pathology, Cancer Institute Hospital, Tokyo, Japan; 5 Department of Diagnostic Pathology, Kameda Kyobashi Clinic, Tokyo, Japan; 6 Department of Breast Oncology, Aichi Cancer Center Hospital, Nagoya, Japan; 7 Department of Breast Surgery, Toranomon Hospital, Tokyo, Japan; 8 Department of Breast Medical Oncology, Cancer Institute Hospital, Tokyo, Japan; 9 Department of Breast Oncology, National Hospital Organization Shikoku Cancer Center, Matsuyama, Japan; 10 Department of Breast Surgery Oncology, St Lukes International Hospital, Tokyo, Japan; 11 Department of Breast and Medical Oncology, National Cancer Center Hospital East, Kashiwa, Japan; 12 Department of Surgery, Breast Oncology, National Hospital Organization Osaka National Hospital, Osaka, Japan

**Keywords:** Breast cancer, Neoadjuvant chemoradiotherapy, Pathological therapeutic effect, Specimen sampling method

## Abstract

**Background:**

In JCOG0306 trial, a phase II study to examine the efficacy of neoadjuvant chemotherapy followed by radiation therapy (NAC-RT) to primary breast cancer, pathological complete response (pCR) was evaluated from specimens of the representative cross-section including the tumor center that had been accurately marked [representative specimen (RS) method]. In this ancillary study, we examined if the RS method was comparable to the conventional total specimen (TS) method, which is widely employed in Japan, to identify the pCR group showing excellent prognosis.

**Methods:**

We obtained long-term follow-up data of 103 patients enrolled in JCOG0306 trial. As histological therapeutic effect, pCR (ypT0 and ypT0/is) and quasi-pCR [QpCR, ypT0/is plus Grade 2b (only a few remaining invasive cancer cells)] were evaluated with RS and TS methods. Concordance of pCR between these two methods and associations of the pCR with prognosis were examined.

**Results:**

ypT0, ypT0/is, and QpCR were observed in 28 (27.2%), 39 (37.9%), and 45 (43.7%) patients with RS method, whereas these were 20 (19.4%), 25 (24.3%) and 40 (38.9%) with TS method, respectively. Between RS and TS methods, concordance proportions of ypT0 and ypTis were 92.2% and 86.4%, respectively. Risk of recurrence of ypT0/is group was lower than that of non-ypT0/is group (HR 0.408, 95% CI [0.175–0.946], *P* = 0.037) and risk of death of ypT0/is group was lower than that of non-ypT0/is group (HR 0.251, 95% CI [0.073–0.857], *P* = 0.027). The ypT0 and ypT0/is groups with RS method showed excellent prognosis similarly with those with TS method, and RS method was able to differentiate the OS and RFS between pCR and non-pCR than TS method significantly even if pCR was classified ypT0 or ypT0/is. With TS method, QpCR criteria stratified patients into the better and worse prognosis groupsmore clearly than pCR criteria of ypT0 or ypT0/is.

**Conclusions:**

RS method was comparable to TS method for the evaluation of pCR in the patients who received NAC-RT to primary breast cancer provided the tumor center was accurately marked. As pCR criteria with RS method, ypT0/is appeared more appropriate than ypT0.

**Supplementary Information:**

The online version contains supplementary material available at 10.1007/s10549-024-07408-5.

## Background

Neoadjuvant therapy to the patients with primary breast cancer is widely used for the purpose of down-staging of disease prior to breast conservation therapy and for the evaluation of sensitivity of the cancer cells to the therapy [1, 2]. Therapeutic effect is evaluated by histopathological examination of the resected specimens, and the diagnosis of pathological complete response (pCR) is accepted as a good indicator of better prognosis [3, 4]. pCR has often been employed as the primary endpoint of clinical trials of primary systemic therapies [5, 6]. Whether the effect is pCR or non-pCR is also very important to decide the following treatment plan in recent routine practice [7, 8].

Since the initial pCR criteria of the National Surgical Adjuvant Breast and Bowel Project (NSABP) B-18 trial [9], multiple criteria for pCR were proposed, and recent consensus of pCR appears being fixed as no residual carcinoma (ypT0) or no residual invasive carcinoma component with residual non-invasive carcinoma component (ypTis) in the primary site (ypT0/is) [10, 11]. It is undetermined if axillary lymph node status is included in the evaluation of pCR, although recent trend of clinical trials tends to define pCR as ypT0/is ypN0 [6, 8]. In addition, the best specimen sampling method is also undetermined for the evaluation of pathological therapeutic effect in daily practice or clinical trials [12]. According to recommendations from an international working group, systematic sampling of areas identified by informed mapping of the specimen and close association with clinical and imaging findings is preferable to overly exhaustive sampling and histologic evaluation of the entire tumor bed [total specimen (TS) method] [12, 13]. In Japan, this TS method appears to have been widely employed.

The Japan Clinical Oncology Group (JCOG) trial, JCOG0306, was a single-arm phase II study conducted to examine safety and efficacy of neoadjuvant chemotherapy followed by radiation therapy (NAC-RT) to operable invasive breast cancer conducted at 29 institutions in Japan. pCR was defined as ypT0 or ypTis (ypT0/is) regardless of axillary lymph node status following to criteria of NSABP B-18 [9], and the goal of the trial was a ≧50% of pCR proportion [14]. In JCOG0306 trial, therapeutic effect of NAC-RT to the primary tumor was evaluated by histological examination of only the representative cross-section, including the tumor center (representative specimen [RS] method). However, it remained unclear how response rates and prognosis of responded patients differed between the RS and TS methods.

In the present ancillary study of JCOG0306 (JCOG0306A1), we aimed at examining if the RS method was able to identify pCR patients of better prognosis similarly with the TS method in the same case series. pCR proportions and prognostic significances were also compared between two pCR criteria, i.e., ypT0 and ypT0/is, regardless of nodal status. Because a part of pCR patients according to RS methods may show non-pCR by TS method, the categories of non-pCR, especially the category of only a few remaining invasive cancer cells, were also evaluated for both RS and TS specimens.

## Methods

### Overview of JCOG0306

Key eligible criteria for JCOG0306 are (1) core needle biopsy-proven invasive breast cancer (female only); (2) clinical stage I–IIIA [17]; and (3) tumor diameter of 2–5 cm confirmed by breast ultrasonography [14]. Between June 2004 and April 2005, 108 patients were enrolled. NAC consisted of four courses of doxorubicin 60 mg/m^2^ and cyclophosphamide 600 mg/m^2^ administered intravenously on day 1 every 3 weeks, followed by 12 courses of weekly paclitaxel 80 mg/m^2^. Radiation therapy consisted of a 45 Gy dose administered in 25 fractions to the whole breast over 5 weeks using tangential fields, followed by a 10 Gy boost in 5 fractions over 1 week to the original tumor region. After completion of NAC-RT, surgical therapy was performed for 106 patients: partial resection for 97 and mastectomy for 9. The proportion of breast conserving surgery was 88.9% (96/108). In the final report, pCR (ypT0/is ypN any) proportion by NAC-RT in the 108 patients was 36%, and 4 year recurrence-free survival (RFS) and overall survival (OS) were 84.1% and 93.5%, respectively [14].

***Ethical issues.***

The protocol of this ancillary study was reviewed and approved by the JCOG Protocol Review Committee and the institutional review board of each participating institution.

### Pathology specimen sampling

In JCOG0306 trial, tumor center was determined before NAC-RT and marked on the translucent overhead projector sheet that covered the affected breast. On surgery, the tumor center was re-identified by covering the sheet on the breast and according to the marked tumor center, the representative cross-section was determined, and therapeutic effect was evaluated for the slides of the representative cross-section only (RS method). In parallel with the representative cross-section, the specimen was cut into 5–10 mm thick slices.

**Fig. 1:**
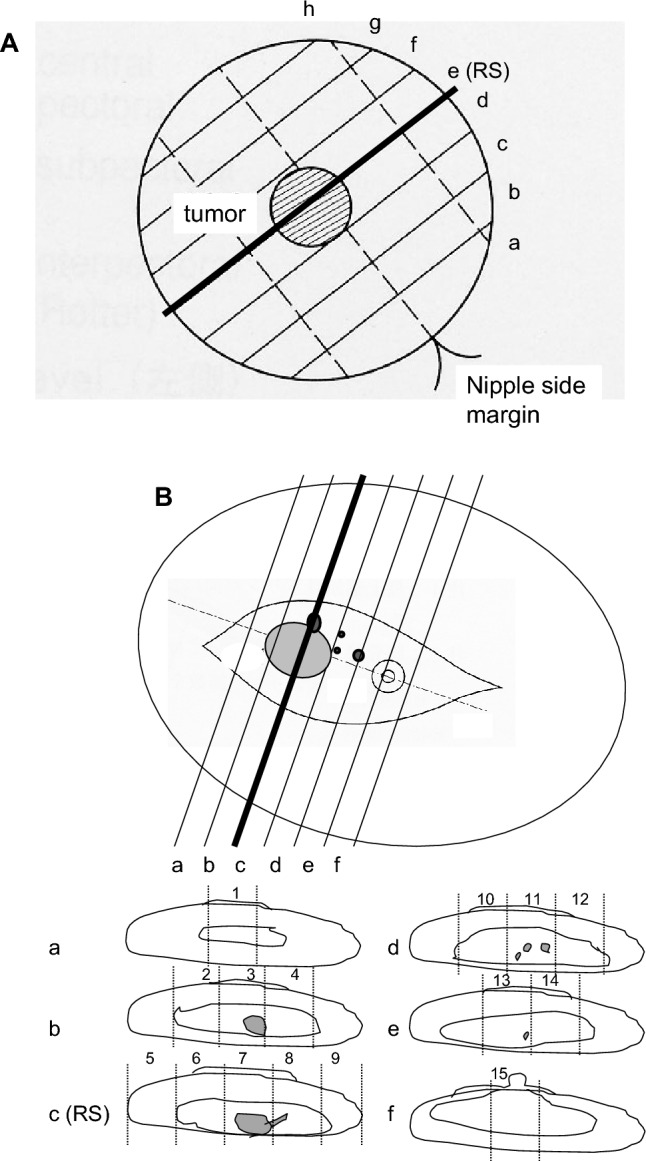
Schematic presentation of surgically resected specimens and sampling. **A** A partial resection specimen. Tumor center was marked before NAC-RT and according to the marking, representative cross-section including the tumor center was sampled for central pathology review (CPR) in JCOG0306 trial (RS method). Then, the specimens were cut into 5–10 mm thick slices, and all these slices were sampled for tissue slides [15]. In this study, all sampled slides were evaluated (TS method). **B** A mastectomy specimen with high probability of pCR. Tissue blocks were sampled from the sections that contain the whole area of tumor bed (dark gray), including the representative cross-section sampled with RS method. In this case, 15 tissue blocks were sampled. The therapeutic effect was CPR for section **c** in JCOG0306 trial, but in this study all samples (Sections **a**–**f**) were evaluated

In total specimen (TS) method for a partial resection specimen, tissue sections were made from the blocks of the entire specimen according to the General Rules for Clinical and Pathological Recording of Breast Cancer, 14th ed., Japan (abbreviated as General Rules) [15] (Fig. 1a). In TS method for a mastectomy specimen, tissue sections were made from the area including whole tumor bed where a tumor was and/or had been located in addition to the representative cross-section (Fig. 1b). In JCOG0306A1, the therapeutic effect was studied for all these specimens processed with TS method.

### Histopathological evaluation

In JCOG0306, according to the criteria of NSABP B-18, pCR was defined as disappearance of invasive carcinoma component (ypT0/is) regardless of lymph node status [9]. In the present study, two criteria for pCR were considered: (1) ypT0 (no residual carcinoma) and (2) ypT0/is (no residual invasive carcinoma component with or without residual non-invasive carcinoma component).

Tumors with residual invasive carcinoma component (non-pCR) were also classified by the General Rules into grades 0, 1a, 1b, 2a, and 2b [15]. Grade 0, 1a, 1b, and 2a were defined as almost no changes, mild changes regardless of the extent and/or marked changes in < 1/3 of cancer cells, marked changes in ≥ 1/3– < 2/3 of cancer cells, and marked changes in ≥ 2/3 of tumor cells with apparent remaining cancer cells, respectively [16]. Grade 2b was defined as marked changes showing almost complete response with only a few remaining cancer cells. Grade 2b effect and the category of combination of pCR and grade 2b (ypT0/is/Grade 2b), named quasi-pCR (QpCR), were shown to be associated with good prognosis equally to pCR in several clinical studies [17, 18]. In this study, a part of patients evaluated as ypT0 or ypT0/is with RS method were expected to contain a few (grade 2b) or apparent (Grade 0/1a/1b/2a) remaining invasive cancer cells by the examination with TS method. Therefore, non-pCR was dichotomized into grade 2b and grade 0/1a/1b/2a, and prognostic impact of QpCR was also evaluated with both RS and TS methods.

In JCOG0306 trial and the present study, histopathological therapeutic effect was evaluated independently among three central pathology review (CPR) panels (MK, FA, HT), and the majority diagnosis was adopted as the final judgments. When the diagnosis differed among three panels, consensus was acquired over a discussion microscope. Information of axillary lymph node metastasis status in the resected specimen was collected from JCOG0306.

### Immunohistochemistry (IHC)

Estrogen receptor (ER) and progesterone receptor (PgR) statuses were determined by IHC at each institute. Tumors with > 10% of tumor cells showing positive staining were classified as positive for ER and PgR [19]. Human epidermal growth factor receptor 2 (HER2) status was also determined at each institute with IHC or fluorescence in situ hybridization (FISH) and 3+ on IHC or positive with FISH was defined a positive [20].

### Statistical analysis

We analyzed the concordance proportions of pCR (ypT0, ypT0/is) between RS and TS methods for the primary breast tumor in 103 patients. RFS and OS curves were drawn using Kaplan–Meier method. Hazard ratio (HR) and 95% confidence interval (CI) were calculated using the Cox univariable analyses. In accordance with JCOG0306, nodal status was not considered for survival analyses. Two-sided* p* value of < 0.05 was defined statistically significant. All statistical analyses were conducted by SAS 9.2 or later.

## Results

### Comparison of pCR proportion between RS and TS methods

In this study, hematoxylin–eosin stained pathology slides of surgically resected specimens were available from 103 patients. Specimens from 3 patients were not available: Two patients were diagnosed as DCIS by CPR for core needle biopsy specimens prior to NAC-RT, and review of slides was not possible for another patient.

For the 103 patients, further follow-up data were obtained in each institution, and median follow-up period was 11.6 years. Median patient age was 51 (from 23 to 69). Clinical stages prior to NAC-RT were I, IIA, IIB and IIIA in 1, 50, 48, and 4, respectively, 63 being ER positive and 33 HER2 positive (Table 1).

**Table 1:** Consensus diagnosis of primary breast tumor obtained by core needle biopsy before treatment by central pathology review

Factors	Number of patients (%)
Age (year)	
Median (range)	51 (23–69)
Menopause	
Pre	54 (52.4)
Post	49 (47.6)
Histology	
Invasive ductal	96 (93.2)
Invasive lobular	4 (3.9)
Mucinous	2 (1.9)
Apocrine	1 (1.0)
Histological grade^a^	
Grade 1	18 (17.5)
Grade 2	31 (30.1)
Grade3	26 (25.2)
Unknown	22 (21.3)
cT	
T1	1 (1.0)
T2	99 (96.1)
T3	3 (2.9)
cN	
N0	52 (50.5)
N1	49 (47.6)
N2	2 (1.9)
Estrogen receptor	
Positive	63 (61.2)
Negative	38 (36.9)
Unknown	2 (1.9)
Progesterone receptor	
Positive	53 (51.5)
Negative	48 (46.6)
Unknown	2 (1.9)
HER2	
Positive	33 (32.0)
Negative	67 (65.0)
Unknown	3 (2.9)
Surgery	
Mastectomy	8 (7.8)
Partial mastectomy	95 (92.2)

^a^Histological grade was given in invasive ductal carcinoma and apocrine carcinoma

With RS method, ypT0, ypTis, Grade 2b, and Grade 0/1a/1b/2a were observed in 28, 11, 6, and 58 patients. ypT0 and ypT0/is were observed in 28 (27.2%) and 39 (37.9%) patients, in whom 27 (26.2%) and 37 (35.9%) were ypN0, respectively (Table 2).

**Table 2:** Comparison of histopathological therapeutic effect between the evaluations with representative specimen (RS) and total specimen (TS) methods

Therapeutic effect	Subtotal	Number of cases (%)			
		TS method			
		ypT0	ypTis	Grade 2b	Grade 0,1a,1b,2a
RS method					
ypT0	28	19 (68)	2 (74)	5 (18)	2 (7)
ypTis	11	1 (9)^a^	3 (27)	5 (46)	2 (18)
Grade 2b	6	0 (0)	0 (0)	4 (67)	2 (33)
Grade 0, 1a, 1b, 2a	58	0 (0)	0 (0)	1 (2)	57 (98)
	103	20	5	15	63

^a^In one patient, CPR diagnosis was ypTis for RS but was ypT0 for TS. By the review, only a small number of highly degenerated atypical cells were seen in the specimen but the opinion of the panels had been split between ypT0 and Grade 2b in JCOG0306 trial (RS evaluation), and the consensus of Grade 2b had been fixed. In re-evaluation for TS, conducted independently of the former RS evaluation, the consensus was ypT0

With TS method, ypT0, ypTis, Grade 2b, and Grade 0/1a/1b/2a were observed in 20, 5, 15 and 63 patients. ypT0 and ypT0/is were observed in 20 (19.4%) and 25 (24.3%) patients, in whom 19 (18.4%) and 23 (22.3%) were ypN0, respectively (Table 2).

### Concordance proportions between RS and TS methods

The concordance proportions of ypT0 and ypT0/is between RS and TS methods were 90.3% (93/103) and 86.4% (89/103), respectively. Against the therapeutic effect of ypT0 with TS method, the sensitivity and specificity of ypT0 with RS method were 95.0% (19/20) and 89.2% (74/83), respectively. Likewise, the sensitivity and specificity of ypT0/is with RS method were 100% (25/25) and 82.1% (64/78), respectively. Photomicrographs of primary tumors that showed concordant and discordant therapeutic effects between RS and TS method are presented in Supplementary Figs. 1, 2.

The down-grade proportions of therapeutic effects from pCR with RS method to non-pCR with TS method were as follows: In 9 (32%) of 28 ypT0 cases with RS method, 2, 5, and 2 were down-graded as ypTis Grade 2b and Grade 2a with TS method, respectively. In 7 (64%) of 11 ypTis cases, 5 and 2 were down-graded as Grade 2b and Grade 2a with TS method, respectively. Therefore, down-grade proportions from pCR with RS method to non-pCR with TS method tended to be higher in ypTis (7/11, 64%) than in ypT0 (9/28, 32%) in ypT0.

### Prognostic significance of pCR with RS method

**Fig. 2:**
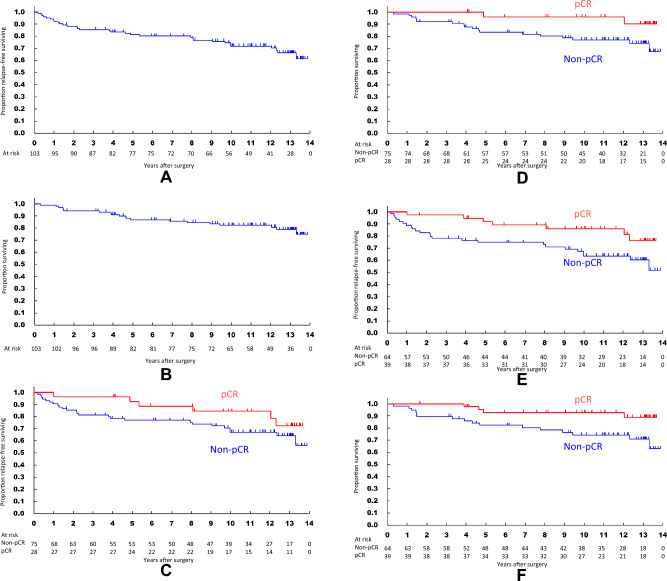
Survival curves evaluated with representative specimen (RS) method. **A** The recurrence-free survival (RFS) curve for all 103 patients. **B** The overall survival (OS) curve for all 103 patients. **C** RFS curves for ypT0 group (*n* = 28) and the other group (*n* = 75). **D** OS curves for ypT0 and the other groups. **E** RFS curves for ypT0/is group (*n* = 39) and the other group (*n* = 64). **F** OS curves for ypT0/is and the other groups

10 yr-RFS and 10 yr-OS in 103 patients were 71.8% and 82.3%, respectively. RFS and OS curves are shown in Fig. 2A, B. Detailed values of survival analyses are presented in Supplementary Table 1.

With RS method, RFS curves did not differ significantly between ypT0 group (*n* = 28) and non-ypT0 group (*n* = 75) with 10 years RFS rates of 84.5% and 67.2%, respectively (Fig. 2C). Risk of recurrence by Cox univariable analysis tended to be lower in ypT0 group, but the difference was not significant (HR 0.543, 95% CI [0.22–1.325], *P* = 0.179). OS curves differed between ypT0 and non-ypT0 groups with 10 years OS of 96.2% and 77.1%, respectively (Fig. 2D). The risk of death of ypT0 group tended to be lower than that of non-ypT0 group (HR 0.244, 95% CI [0.057–1.056], *P* = 0.059).

With RS method, RFS curves differed between ypT0/is group (*n* = 39) and non-ypT0/is group (*n* = 64) with 10 years RFS of 86.1% and 63.4%, respectively (Fig. 2E). Risk of recurrence of ypT0/is group was lower than that of non-ypT0/is group (HR 0.408, 95% CI [0.175–0.946], *P* = 0.037). OS curves also differed between these two groups with 10 years OS of 94.6% and 74.9%, respectively (Fig. 2F). The risk of death of ypT0/is group was lower than that of non-ypT0/is group (HR 0.251, 95% CI [0.073–0.857], *P* = 0.027).

### Prognostic significance of pCR with TS method

**Fig. 3:**
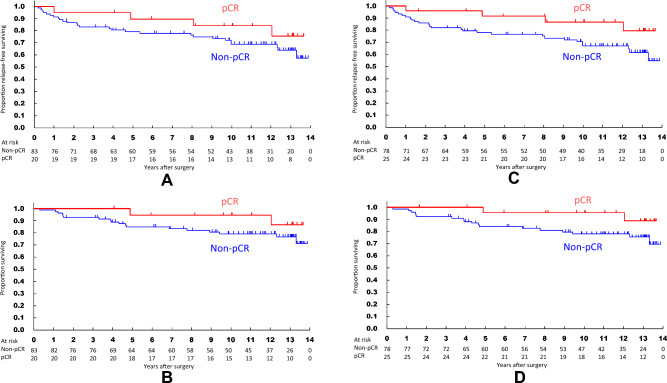
Survival curves evaluated with total specimen (TS) method. **A** Recurrence-free survival (RFS) curves for ypT0 group (*n* = 20) and the other group (*n* = 83). **B** Overall survival (OS) curves for pT0 and the other groups. **C** RFS curves for ypT0/is group (*n* = 25) and the other group (*n* = 78). **D** OS curves for ypT0/is and the other groups

With TS method, RFS curves tended to differ between the ypT0 (*n* = 20) and non-ypT0 (*n* = 83) groups with 10 years RFS of 84.1% and 68.9%, respectively (Fig. 3A). Recurrence risk of ypT0 also tended to be lower than that of non-ypT0 group (HR 0.527, 95% CI [0.184–1.507], *P* = 0.232). There were no significant differences in OS curves between these two groups with 10 years OS of 94.7% and 79.3%, respectively (Fig. 3B). Risk of death of ypT0 group tended to be lower but did not differ significantly from non-ypT0 group (HR 0.389, 95% CI [0.090–1.680], *P* = 0.206).

With TS method, RFS curves tended to differ between the ypT0/is (*n* = 25) and non-ypT0/is group (*n* = 78) with 10 years RFS of 86.8% and 67.2%, respectively **(**Fig. 3C**)**. Recurrence risk of ypT0/is tended to be lower than that of non-ypT0/is group (HR 0.399, 95% CI [0.140–1.142], *P* = 0.087). There was no significant difference in OS curves between these two groups with 10 years OS of 95.7% and 78.1%, respectively **(**Fig. 3D**)**. Risk of death of ypT0/is group tended to be lower but did not differ significantly from non-ypT0/is group (HR 0.302, 95% CI [0.070–1.301], *P* = 0.108).

### Significance of QpCR with RS and TS methods: supplementary analysis

With RS and TS methods, QpCR was observed in 45 (43.7%) and 40 (38.9%), in whom 42 (40.8%) and 38 (36.9%) were ypN0, respectively. The sensitivity and specificity of QpCR with RS method against QpCR with TS method were 98% (39/40) and 90% (57/63), respectively. The down-grade proportions from QpCR with RS method to Grade 0/1a/1b/2a residual carcinoma with TS method were 7% (2/28) in ypT0, 18% (2/11) in ypTis, and 33% (2/6) in Grade 2b (Table 2).

With RS method, RFS curves differed between QpCR group (*n* = 45) and non-QpCR group (*n* = 58) with 10 years RFS of 82.4% and 63.9%, respectively. Recurrence risk of QpCR was lower than that of non-QpCR group (HR 0.461, 95% CI [0.212–1.001], *P* = 0.050). OS curves also differed between these two groups with 10 years OS of 92.8% and 74.4%, respectively (Supplementary Fig. 3A, B). The risk of death of QpCR was lower than that of non-QpCR group (HR 0.283, 95% CI [0.095–0.847], *P* = 0.024).

With TS method, RFS curves differed between QpCR (*n* = 40) and non-QpCR (*n* = 63) groups with 10 years RFS of 89.2% and 60.8%, respectively. Recurrence risk of QpCR group was lower than that of non-QpCR group (HR 0.306, 95% CI [0.125–0.748], *P* = 0.009). OS also differed between these two groups with 10 years OS being 94.6% and 74.5%, respectively (Supplementary Fig. 3C, D). The risk of death of QpCR group was lower than that of non-QpCR group (HR 0.238, 95% CI [0.070–0.811], *P* = 0.022) (Supplementary Table 1).

## Discussion

Currently, escalation and de-escalation are applied to optimize treatment strategies for breast cancer patients. Both local and intensive systemic treatments are recommended for early breast cancer patients at high risk of recurrence, while those at low risk should receive less intensive therapy. An accurate assessment of biological property of the tumor is needed for selection of treatment strategies not only in adjuvant therapy but also in primary systemic therapy. For example, the CREATE-X trial showed that adjuvant capecitabine improved the outcomes of patients with residual invasive disease after NAC [7]; The KATHERINE trial demonstrated that post-NAC clinical outcome of adjuvant trastuzumab emtansine after surgery for patients with residual invasive disease was better than that of trastuzumab alone [8]. To determine the adjuvant therapy, it is important to consider the evaluation methods for pCR after NAC.

To accurately evaluate pCR in surgically resected specimens, it seems better to examine therapeutic effect as much as possible by sampling the whole-resected specimens as TS method. However, from practical viewpoint, minimally required specimen sampling from the resected tissues, e.g., RS method, would be more reasonable so long as pCR with RS method is equal to pCR with TS method in terms of pCR proportions and long-term patient prognosis.

In the present study, we at first compared pCR proportions between RS and TS methods and analyzed histological features of discrepant cases. Sixteen (41%) of 39 pCR (ypT0/is) cases (9 of 28 ypT0 and 7 of 11 ypTis) with RS method were down-graded as non-pCR with TS method. It was of note that 10 (63%) of these 16 ypT0/is cases were Grade 2b with TS method, and such discrepancy was more frequent in ypTis than in ypT0 cases. Then, survival analyses were conducted. 10 years RFS and OS curves of pCR and non-pCR groups, based on both ypT0 and ypT0/is criteria, were shown to be almost similar between RS and TS methods. However, RS method was able to differentiate the OS and RFS between pCR and non-pCR than TS method significantly even if pCR was classified ypT0 or ypT0/is (Supplementary Table 1). Considering both the practical viewpoint and the possibility to stratify prognosis, RS method can be reasonable. In addition, ypT0/is criteria more significantly stratified patients into better and worse prognosis groups than ypT0 criteria by RS methods. Therefore, ypT0/is criteria were considered to identify pCR group than ypT0 criteria more effectively with RS method and appeared worth considering introduction in routine practice or clinical trials. The RS method used in JCOG0306 was conducted under strict protocol of tumor center orientation and identification of the representative cross-section. This point should be considered to avoid overlooking Grade 0/1a/1b/2a residual disease when the application of RS method is considered.

Third, we examined prognostic impact of QpCR (ypT0/is + Grade 2b). QpCR with RS method could also classify the patient group of excellent long-term prognosis for both RFS and OS. However, 10 years RFS of QpCR group appeared somewhat lower than that of ypT0/is, and down-grade proportion of Grade 2b with RS method to Grade 0/1a/1b/2a with TS method was 33%, which was higher than 18% in ypTis and 7% in ypT0. From these results, it was considered that QpCR is not a good indicator of better prognosis after NAC-RT with RS method, and there is also a possibility that residual lesions are not adequately evaluated by RS method. Therefore, ypT0/is was considered to be better than QpCR.

On the other hand, QpCR with TS method (*n* = 40) could stratify most clearly the 103 patients into low and high-risk groups. With TS method, the number of QpCR cases was larger than ypT0 or ypT0/is cases, and RFS and OS of non-QpCR group were lower than those of non-ypT0 and non-ypT0/is groups (Supplementary Table 1). QpCR group appeared to pick up the patients of excellent prognosis who were overlooked by ypT0 or ypT0/is criteria and a majority of these patients showed Grade 2b effect. In other words, these results suggest that so long as TS method is introduced, it was probable that grade 2b effect picked up minute residual carcinoma cells that have little influence on worse prognosis.

NAC with molecular targeted therapy is well known to be effective and to dramatically improve pCR proportions (about 63%) in the patients with HER2-positive breast cancer. The high probability of pCR suggests that omitting surgery is an effective alternative to resection in breast cancer patients showing possibly pCR in response to NAC. In fact, clinical trials to validate this strategy are now enrolling patients [21–24]. In these trials, surgery is omitted, and radiation is administered after systemic therapy. We need to evaluate the residual cancer cells after both systemic and radiation therapy with these treatment strategies. However, data pertaining to pathological evaluation for core needle biopsy or vacuum-assisted biopsy after NAC-RT are yet lacking. The present study was a good model of this concept and supported that a labor-saving specimen sampling method can be worth studying for evaluation of pCR status in these biopsy specimens.

Limitations in this study consist in several points. First, the number and events of each subgroup of ypT0, ypTis, and Grade 2b was so small that statistical power to detect each prognostic impact was not sufficient. Nonetheless, it was possible to suggest both ypT0 and ypT0/is with RS method was appropriate to evaluate therapeutic effect of NAC-RT. Second, the definition of Grade 2b is not quantitatively determined well except for “only a few remaining invasive cancer cells.” A lot of studies support the idea that Grade 2b was a good prognostic indicator as near pCR [17, 18], but another report argues that the survival curve for Grade 2b group was close to that for Grade 2b group rather than pCR group [25]. Therefore, a further study to establish the quantitative criteria of Grade 2b is necessary for a larger number of cases.

Third we did not measure residual cancer burden (RCB) because most of the data were acquired by CPR during the period of JCOG0306, and we have no idea to co-evaluate RCB at that time [26–28]. We plan to perform a novel study to establish Grade 2b criteria and to compare those with RCB in a larger number of cases.

## Conclusion

pCR with RS method was comparable to pCR with TS method and especially ypT0/is criteria with RS method appeared to be applicable for the evaluation of pCR in the patients who received NAC-RT to primary breast cancer provided the tumor center was accurately marked.

## Supplementary Information

Below is the link to the electronic supplementary material.

Supplementary file1 (DOCX 1329 kb)

## Data Availability

The data that support this study's findings are available from JCOG, but restrictions apply to their availability. These data were used under license for the current study and are not publicly available. Data are, however, available from the authors upon reasonable request and with permission of JCOG.

## Abbreviations

CI: Confidence interval
CPR: Central pathology review
ER: Estrogen receptor
FISH: Fluorescence in situ hybridization
HER2: Human epidermal growth factor receptor 2
HR: Hazard ratio
JCOG: Japan Clinical Oncology Group
NAC-RT: Neoadjuvant chemotherapy followed by radiation therapy
NSABP: National Surgical Adjuvant Breast and Bowel Project
pCR: Pathological complete response
PgR: Progesterone receptor
RFS: Recurrence-free survival
RS: Representative specimen
TS: Total specimen
