# Supplementary material for: Comparison of proportions and prognostic impact of pathological complete response between evaluations of representative specimen and total specimen in primary breast cancer after neoadjuvant chemoradiotherapy: an ancillary study of JCOG0306
Source: Breast Cancer Res Treat. 2024 Jun 27;208(1):145–54. doi: 10.1007/s10549-024-07408-5 (PMC11452473; doi:10.1007/s10549-024-07408-5)

Supplementary Table 1 Comparisons of survival proportions and hazard ratios of patient groups striated by ypT0, ypT0/is, and QpCR criteria

Therapeutic No. of 10-yr RFS HR 10yr OS HR

Effect patients % (95% CI) (95% CI) % (95% CI) (95% CI)

1. RS method

ypT0 28 84.5 (63.7–93.9) 0.543  **96.2 (75.7**–**99.4)**  0.244

Non-ypT0 75 67.2 (54.6–76.9) (0.222–1.325) **77.1 (65.2**–**85.3)** (0.057–1.056)

*P* value 0.180 0.059

**ypT0/is 39 86.1 (69.8**–**94.0) 0.408 94.6 (80.0**–**98.6) 0.251**

**Non-ypT0/is 64 63.4 (49.7**–**74.3) (0.175**–**0.946) 74.9 (61.7**–**84.1) (0.073**–**0.857)**

*P* value **0.037** **0.027**

**QpCR 45 82.4 (66.3**–**91.3)**  0.461 **92.8 (79.4**–**97.6) 0.283**

**Non-QpCR 58 63.9 (49.7-75.1)** (0.212–1.001)  **74.4 (60.5**–**84.0) (0.095**–**0.847)**

*P* value 0.050 **0.024**

1. TS method

ypT0 20 84.1 (58.3–94.6) 0.527 94.7 (68.1–99.2) 0.389

Non-ypT0 83 68.9 (57.1–78.1) (0.184–1.507) 79.3 (68.3–86.8) (0.090–1.680)

*P* value 0.232 0.206

ypT0/is 25 86.8 (64.3–95.6) 0.399 95.7 (72.9–99.4) 0.302

Non-ypT0/is 78 67.2 (54.9–76.8) (0.140-1.142) 78.1 (66.6–86.0) (0.070–1.301)

*P* value 0.087 0.108

**QpCR 40 89.2 (73.7**–**95.8) 0.306 94.6 (80.1**–**98.6) 0.238**

**Non-QpCR 63 60.8 (46.9**–**72.2) (0.125**–**0.748) 74.5 (61.2**–**83.8) (0.070**–**0.811)**

*P* value **0.009** **0.022**

*CI* confidence interval, *HR* hazard ratio, *OS* overall survival, *QpCR* quasi-pathological complete response, *RFS* recurrence-free survival, *RS* representative specimen, *TS* total specimen

Supplementary Figure 1 Photomicrographs of the tumors that showed consistent therapeutic effect between representative specimen (RS) and total specimen (TS) methods. **A** and **B** A case showed ypT0 in both RS (**A**) and TS (**B**) methods. Foamy macrophages containing hemosiderin are seen. **C** and **D** Another case showing ypTis with both RS (**C**) and TS (**D**) methods. Ductal carcinoma in situ component with mild degenerative changes are seen. **E** and **F** Another case showing Grade 2b with both RS (**E**) and TS (**F**) methods. A small number of degenerated invasive carcinoma cells are seen (arrows)

Supplementary Figure 2 Photomicrographs of the tumors that showed inconsistent therapeutic effect between representative specimen (RS) and total specimen (TS) methods. **A** and **B** A case showing ypT0 with RS method (**A**) but residual invasive carcinoma (Grade 2a) with TS method (**B**). **C** and **D** Another case showing ypT0 with RS method (**C**) but non-pCR (Grade 2a) with TS method (**D**). Fibrosis and normal mammary gland (arrows) are seen in (**C**), but viable residual invasive carcinoma cells are seen in (**D**) (arrows). **E** and **F** A case showing Grade 2b with RS method (**E**), but Grade 2a with TS method (**F**). A small number of degenerated residual invasive carcinoma cells are seen in (**E**) (inset), but more massive viable carcinomas remain in (**F**) (inset)

Supplementary Figure 3 Survival curves of QpCR and the other groups evaluated with representative specimen (RS) and total specimen (TS) methods. **A** Recurrence-free survival (RFS) curves for QpCR (*n* = 45) and the other (*n* = 58) groups evaluated with RS method. **B** overall survival (OS) curves for QpCR and the other groups evaluated with RS method. **C** RFS curves for QpCR (*n* = 40) and the other (*n* = 63) groups evaluated with TS method. **D** OS curves for QpCR and the other groups evaluated with TS method


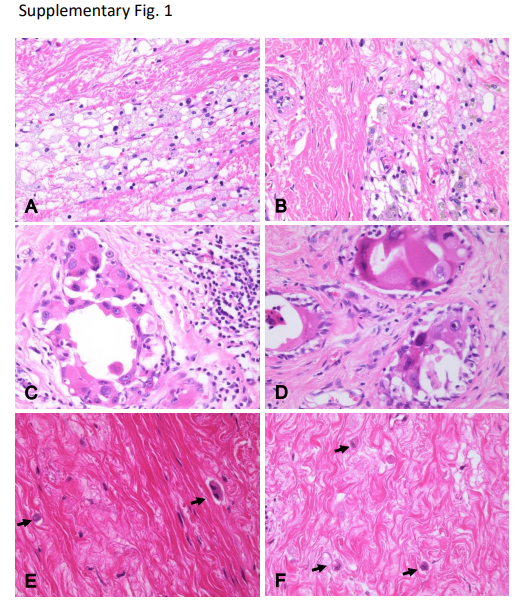


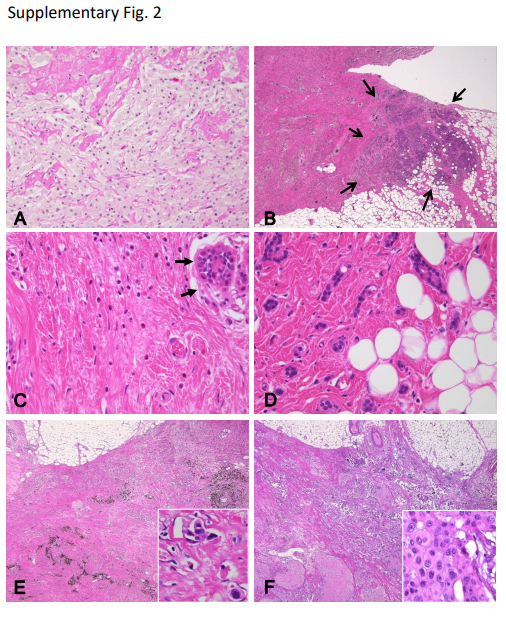


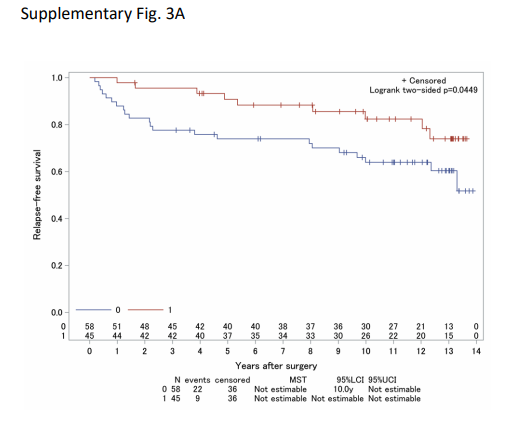


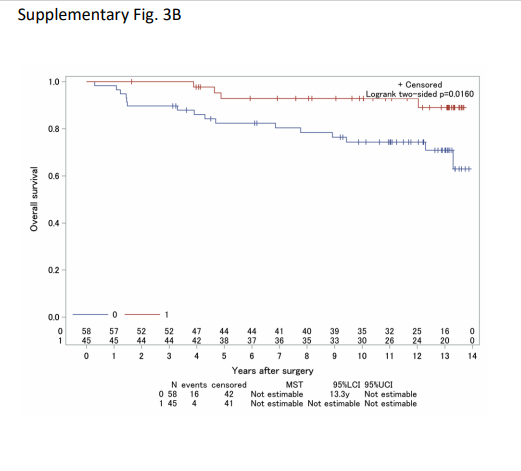


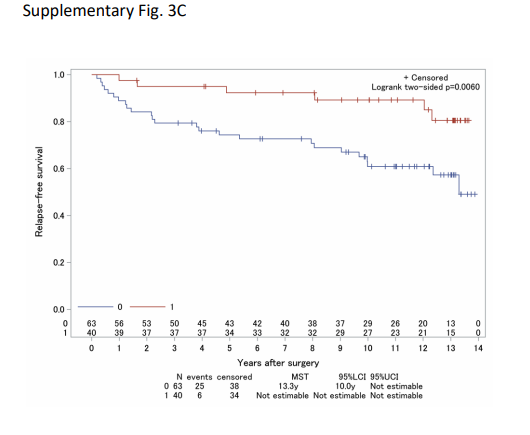
\


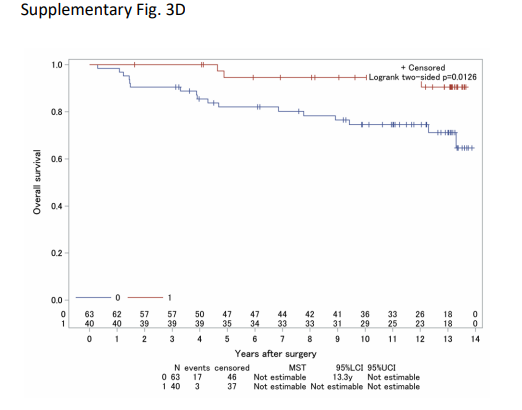

Supplement: Supplementary file 1 — Supplementary file1 (DOCX 1329 kb) [file 10549_2024_7408_MOESM1_ESM.docx]
